# Golden opportunities? How marketing expectations drive purchase intentions of golden rice in Bangladesh and the Philippines

**DOI:** 10.1080/21645698.2024.2418161

**Published:** 2024-11-18

**Authors:** Dilshad Zahan Ethen, Maimuna Begum, Berre Deltomme, Md. Rasheduzzaman, Josefina F. Ballesteros, Riza Abilgos-Ramos, Mohammad Jahangir Alam, Alice Onek Atimango, Hans De Steur

**Affiliations:** a Department of Agricultural Economics, Faculty of Bioscience Engineering, Ghent University, Gent, Belgium; b Department of Agribusiness and Marketing, Bangladesh Agricultural University, Mymensingh, Bangladesh; c Department of Agricultural Marketing and Business Management, Sylhet Agricultural University, Sylhet, Bangladesh; d Agricultural Economics Division, Bangladesh Agricultural Research Institute, Gazipur, Bangladesh; e Senior Economic development Specialist, National Economic and Development Authority (NEDA), Eastern Visayas, Philippines; f Science Research Specialist, Philippine Rice Research Institute (PhilRice), Science City of Muñoz, Nueva Ecija, Philippines; g Department of Rural Development and Agribusiness, Faculty of Agriculture and Environment, Gulu University, Gulu, Uganda

**Keywords:** Bangladesh, consumer expectations, expectation confirmation theory, golden rice, purchase intentions, structural equation modeling, the Philippines

## Abstract

Golden Rice (GR), genetically modified (GM) rice enriched with provitamin A, holds promise to address micronutrient deficiencies in developing countries. However, its success hinges on market acceptance. This study investigates how the marketing aspects of GR influence consumers’ purchase intentions in Bangladesh and the Philippines. The Expectation Confirmation Theory (ECT) is employed to analyze the role of expectations regarding the marketing mix components (i.e. product, price, place, promotion), risk perceptions, performance expectations, and expected satisfaction on consumers’ purchase intentions. Data from online surveys in Bangladesh (*n* = 391) and the Philippines (*n* = 354), collected using convenience sampling, were analyzed using structural equation modeling. Findings reveal that positive expectations toward the marketing mix, performance, and satisfaction increase consumers’ purchase intention of GR, whereas risk perceptions have a negative influence. Additionally, it was found that expectations toward all four marketing mix components significantly affect purchase intention in Bangladesh. However, only product and promotion have a notable influence in the Philippines. These results emphasize the importance of effectively addressing consumers’ marketing expectations to help ensure a successful implementation. This study is novel as it delves into consumers’ purchase intentions for a GM biofortified crop and their expectations for different aspects of its future marketing (i.e. product, price, place, promotion), performance, and satisfaction. If GR is commercialized, future research should validate these expectations based on actual consumer experiences. Additionally, longitudinal studies could track changes in consumer expectations over time, identifying consistently valued marketing elements and offering a valuable technique for product development before launch.

## 1. Introduction

The global population surge and increasing food demands have led to a shift toward advanced agriculture, with a notable focus on innovative technologies such as genetic engineering. This innovative approach, specifically, in developing genetically modified (GM) crops, has played a pivotal role in food security and is now an integral part of sustainable food production systems.^1^ However, recent advancements in biofortification to increase the micronutrient content using several breeding approaches, especially in staples, highlight the necessity of a holistic approach to tackling hunger and poverty.^2^ Solely prioritizing yield and productivity in GM crop development may not be economically viable due to hidden costs associated with nutrient deficiencies.^3^

Micronutrient deficiencies globally burden health, impacting a third of the population.^4^ Common deficiencies like vitamin A, iron, iodine, folate, and zinc can lead to severe conditions, particularly for children and pregnant women in impoverished regions, as highlighted in the Global Nutrition Report.^5^ Vitamin A deficiency (VAD) causes preventable blindness and susceptibility to infections, like diarrheal diseases and measles, affecting over 250 million children worldwide.^6^ About 30% of the children under 5 suffer from VAD globally, with around 2% of the deaths in this group attributed to VAD.^7^ Reliance on rice as a primary food source^8^ leads to inadequate Vitamin A intake in Asian countries like Bangladesh and the Philippines, where rice constitutes up to 76% of the total energy intake.^9^ Consequently, both countries face significant VAD issues, affecting over 41% of children under five in Bangladesh^10^ and 17% of Filipino children aged 6–59 months suffered from VAD in 2018, with the highest prevalence among those aged 12–24 months.^11^

Biofortification of staple crops is a promising approach to enrich the nutrient content of diets and improve human health.^2,12,13^ GM rice enriched with beta- carotene, popularly known as Golden Rice (GR), could fulfill 30–50% of the estimated average daily vitamin A requirement at affordable prices.^14^ However, the introduction of GM biofortified crops like GR faces strong delays due to concerns and controversies surrounding biotechnology.^15,16^ To anticipate its future commercialization in rice-dependent Asian markets, understanding consumer acceptance and expectations is crucial. This is particularly the case for Bangladesh and the Philippines, who both progressed in terms of GR regulation, though at a different pace. While Bangladesh awaits biosafety clearance from its National Committee on Biosafety, the Philippines received approval for commercial propagation of GR in 2021.^17^ However, in April 2024, the Philippine Court of Appeals revoked the permit for the commercial planting of GR in response to a lawsuit filed by Greenpeace and other organizations, despite the aspirations of several officials and scientists in the Philippines to have this rice variety constitute 10% of the nation’s rice harvest within eight years, sufficient to meet the needs of all vitamin A-deficient households in the country.^18^ As such, this ruling may hinder efforts to address VAD in the Philippines and, indirectly, other target regions like Bangladesh.

There is a substantial body of literature measuring consumer acceptance of a wide range of biofortified foods, both conventionally bred and developed through genetic engineering, particularly in developing regions.^3,19–23^ Evidence extensively outlines the importance of the health benefits of GM biofortified crops for consumer acceptance,^24,25^ with a focus on information provision^26,27^ and perceptions about risk and safety.^28,29^ However, these studies typically focus only on product characteristics, illustrating a research gap in how consumers respond to GR marketing.

Prior research on GM biofortified foods often overlooks consumers’ expectations, which is crucial in their purchasing decisions, as consumers actively seek information about packaging, ingredients, prices, and health benefits.^30–32^ Consumers’ perceptions of food quality, particularly with novel technologies like GM, rely on such extrinsic attributes alongside intrinsic attributes.^33^ To evaluate the role of consumer expectations of GR’s marketing mix strategies in shaping purchase intention, we will utilize the marketing mix tool consisting of four main features, namely product, price, place, and promotion. This marketing mix, when well established, can play a pivotal role in influencing consumer decisions.^34^ Additionally, the study also examines the interplay between perceived risks and expectations in driving consumer purchase intentions. Existing research suggests that consumers who are risk-averse are less inclined to purchase GM foods,^35^ especially if they have limited familiarity and knowledge.^36^ Simultaneously, consumers may perceive more significant risks associated with GM crops if they are unaware of the benefits.^37^ Furthermore, negative perceptions regarding various aspects of biofortified GM crops, such as price and environmental impact, can also reduce their acceptance.^38^ Additionally, negative communication about GM crops through media or other channels, such as non-governmental organizations, exacerbates the controversy surrounding GM crops.^39^

This research significantly advances the understanding of consumer evaluation of GM biofortified crops. Firstly, it pioneers the use of a modified Expectation-Confirmation Theory (ECT), extended with the concept of perceived risk, to explain *ex-ante* consumers’ intention to purchase GR. Secondly, it investigates GR marketing expectations and purchase intentions in two highly relevant target countries of GR.^18,40^ As such, this study offers both theoretical and practical insights, allowing to help policymakers, health planners, and marketers to identify strategies that could enhance consumer satisfaction with GR and, hence, improve its potential impact on reducing the burden of VAD.

This issue is particularly relevant to consumers’ intentions to purchase GR, even among urban populations, as a not-yet-commercialized biofortified crop. Although using samples from urban areas limits the generalizability of the results nationwide, this study still provides valuable insights into consumer behavior toward GM biofortified GR. Prior research has indicated that malnutrition and micronutrient deficiencies significantly affect both rural and urban areas in developing regions, necessitating implementing nutrition-sensitive interventions.^19,41^ Current research on GM and other food technologies highlights the importance of understanding the perspectives of young and urban consumers to assess their market potential.^42–46^ Understanding and engaging with the perspectives of these individuals is crucial, as their impact on policy discussions is growing and should be addressed promptly. The importance of educational background in consumer research regarding technology acceptance, particularly among youth, has been highlighted by prior research.^47^ This investigation aims to provide insights from relatively young, educated, and urban consumers who are anticipated to significantly influence the ongoing debate on agricultural biotechnology.

## 2. Current Study

**Figure 1.**
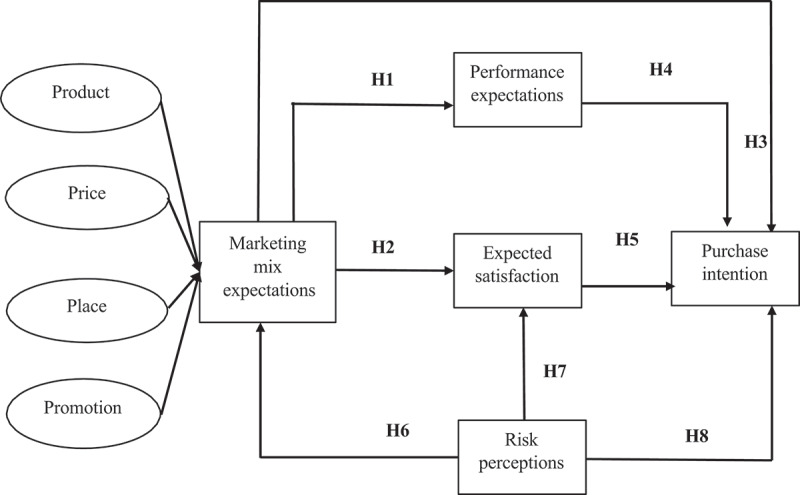
Conceptual framework.

In this study, our goal is to identify the key factors for successfully introducing GR into the markets of the Philippines and Bangladesh. To achieve this, we have utilized the Expectation-Confirmation Theory (ECT), focusing solely on the expectation component. While the ECT is a theory used to understand consumer satisfaction and post-purchase behavior,^48^ we adapted it to investigate the expectations of consumers before the launch of GR, both in terms of performance expectation and expected satisfaction. Additionally, we have considered expectations related to the marketing mix components. Given the common negative perceptions surrounding GM crops due to concerns about their nature, we have also included risk perceptions in our analysis. The outcome variable is the consumers’ purchase intention of GR in this modified ECT. Figure 1 illustrates the proposed model and relationships, leading to the following hypotheses.

H1: Marketing mix expectations positively influence consumers’ performance expectations of GR

H2: Marketing mix expectations positively influence consumers’ expected satisfaction with GR

H3: Marketing mix expectations positively influence consumers’ purchase intention of GR

H4: Performance expectations toward GR positively influence consumers’ purchase intention of GR

H5: Expected satisfaction with GR positively influences consumers’ purchase intention of GR

H6: Risk perceptions about GR negatively influence consumers’ marketing mix expectations of GR

H7: Risk perceptions about GR negatively influence consumers’ expected satisfaction with GR

H8: Risk perceptions about GR negatively influence consumers’ purchase intention of GR

## 3. Methods

### 3.1. Sample & Design

An online survey, created using Qualtrics, was distributed in Bangladesh (*n* = 391) and the Philippines (*n* = 354) using a non-probability (convenience) sampling procedure. The online survey link was distributed to residents of both countries via e-mail and social media (Facebook, Instagram, Messenger). In Bangladesh, 393 consumers received the link between May 18 and June 5, 2021, yielding 391 valid responses after excluding two invalid ones. In the Philippines, a separate link was sent to 501 respondents from October 14 to December 22, 2021, with 354 valid responses after removing 147 incomplete ones. Disparities in internet access due to COVID-19 led to unequal respondent distribution between the two countries. Note that the data collection in the Philippines was conducted after the commercial propagation of GR in June 2021, which was revoked in April 2024 in response to a lawsuit filed by Greenpeace and other organizations. Participation was voluntary, and only fully completed surveys were used in the analysis. The survey consisted of three sections, the first of which presented a concise introduction explaining the study’s purpose, specifying the survey’s duration, emphasizing voluntary participation, and seeking participants’ consent. The second section encompassed the socio-demographic characteristics of the respondents, while the third section delved into consumers’ expectations regarding GR. The survey was pre-tested with a sample of seven students and fifteen rice consumers from diverse backgrounds for each country. This process aimed to identify and resolve any potential issues prior to survey administration, including assessing the clarity and comprehensibility of the questions, the appropriateness of their order, and the necessity of adding or removing certain questions. As the survey was conducted online, it was crucial for the participants to understand each question clearly and answer all the questions with clarity. Based on the participants’ feedback, some questions of the interview schedule were revised, including refining the wording of questions, reassessing their sequence, and improving the overall layout. In this way, the results of the pretest help to develop a standardized interview schedule to ensure the collection of reliable data and to support sound research findings.^49,50^ To counter common method bias (CMB),^51^ preventive measures were implemented by using a note to assure respondents’ anonymity, using pre-validated scales to measure the constructs, employing multiple neutral items, assuring no right or wrong answers, facilitating psychological delineation between the measurement of predictors and criterion variables through their positioning into distinct sections.^52^ The data analysis and result section provides a more detailed explanation of the analytical procedure.

### 3.2. Measures

All items were assessed using a 5-point Likert scale (“1” = strongly disagree, to “5” = strongly agree). Initially, the total number of measurement items for all latent variables in the study was 40 for both countries. However, after conducting exploratory factor analyses (EFAs), six items were removed, leaving 34 items for the final analysis. The phrasing of items was adjusted to fit the study context. First, *marketing mix expectation* was based on four dimensions (i.e., 4 P’s): product, price, place, and promotion, reflecting consumers’ expectations of GR marketing. It was measured through 18 items adapted from existing marketing literature. Six items referred to the “product,”^53–57^ while the other three aspects, namely price,^54,55,58,59^ place,^60^ and promotion,^55^ were each measured by four items. Secondly, *performance expectation* toward GR was measured using four items adapted from previous literature.^61,62^ In this study, it pertains to consumers’ expectations of the performance of GR (e.g., cooking methods, Vitamin A intake level). Thirdly, *consumer satisfaction*, here defined as an ex-ante construct that measured how much GR would satisfy consumers once it becomes available in the market,^63^ was based on four modified items.^64–66^ Fourthly, *risk perceptions* also had four items,^67–70^ similar to *purchase intention*.^35,54,57,71^ Details on all items can be found in Appendix A.

## 4. Data Analysis & Results

### 4.1. Descriptives

Table 1 presents the sociodemographic profile of the respondents for each country. The age distribution in both samples was similar, with the majority being aged between 18 and 30 years old, and only very few above 47 years. There is a gender imbalance, with females dominating in the Philippines (71.5%), but they are less represented in the Bangladesh sample (40.7%). Our sample had more younger respondents, who are adept at using the internet due to their tech-friendliness and extensive online activity.^72^ Unlike older generations, younger individuals often use the internet as their primary source of health information, confidently exploring it to learn new skills and seek awareness.^73^ As for the educational background, the majority of respondents had an undergraduate degree in the Philippines and a post-graduate degree in Bangladesh. Household income showed a significant representation from low- and middle-income groups. As expected, rice was a staple in the diet, with 56% of Bangladeshis consuming it twice daily and 78% of Filipinos eating it three times a day.

**Table 1.** Sample descriptives in Bangladesh and the Philippines.

Measures	Items	Bangladesh (*n* = 391)		The Philippines (*n* = 354)	
		Frequency	%	Frequency	%
Age	Young people (18 to 30)	249	63.7	297	83.9
	Middle-aged people (31 to 46)	128	32.7	47	13.3
	Elderly people (47 to above)	14	3.6	10	2.8
Gender	Male	232	59.3	101	28.5
	Female	159	40.7	253	71.5
Education (years)	Higher secondary	39	10.0	20	5.6
	Under-graduation	92	23.5	247	69.8
	Post-graduation and above	260	66.5	87	24.6
Monthly income	Low	126	32.2	128	36.2
	Medium	80	20.5	142	40.1
	High	185	47.3	84	23.7
Occupation	Unemployed	101	25.8	87	24.6
	Employed (government, private)	247	63.2	224	63.3
	Self-employed	21	5.4	27	7.6
	Others	22	5.6	16	4.5
Marital Status	Unmarried	167	42.7	297	83.9
	Married	224	57.3	57	16.1
Family size	Nuclear Family (≤5 members)	254	65.0	234	66.1
	Extended Family (≥6 members)	137	35.0	120	33.9
No. of children	No of children (0)	172	44.0	182	51.4
	No of children (≤3)	200	51.2	146	41.2
	No of children(≥4)	19	4.9	26	7.3
Place of residence	Urban (e.g., municipal, city, town)	258	66.0	214	60.5
	Semi-urban (e.g., suburb)	85	21.7	48	13.6
	Rural (e.g., village, countryside)	48	12.3	92	26.0
Daily rice consumption frequency	Once	17	4.3	16	4.5
	Twice	217	55.5	62	17.5
	Thrice or above	157	40.2	276	78.0
Awareness of Golden rice	Yes	231	59.1	199	56.2
	No	160	40.9	155	43.8

BD = Bangladesh; PHIL = The Philippines.

Monthly income has been reported in two currencies, namely the Bangladeshi Taka (BDT) and the Philippine peso (PHP).

Monthly income: Low = BD: (1–14,999 BDT); PHIL: (1–15000 PHP); Medium = BD: (15,000 -29,999 BDT); PHIL: (15,001 -30,000 PHP); High = BD: (30,000 BDT to above); PHIL: (above 30, 000 PHP)

In FY 2024, 1 USD = 118 BDT and 1USD = 59 PHP.

Table 2 presents the summary details of the different constructs, which are also abbreviated in the next sections for ease of expression and calculation (the tabulated summary of the measurement items for each latent variable, see Appendix A). The mean values regarding consumers’ expectations toward product, price, and promotion suggest that consumers in the Philippines had, on average, higher expectations compared to those in Bangladesh, except for place expectations, where respondents from both countries had similar expectations. In addition, consumers in the Philippines had slightly higher expectations about the performance of GR compared to those in Bangladesh, while both groups had similar levels of expected satisfaction. Furthermore, the mean score for purchase intention depicts that both groups had fairly high purchase intentions, though slightly higher in Bangladesh, indicating a strong intention to purchase GR in both regions. The mean risk perception score shows that consumers in both regions perceived a relatively low risk associated with GR, with a slightly higher risk perception in the Philippines.

**Table 2.** Summary statistics of the included constructs, mean, and standard deviation.

Variables		Mean (SD)	
		Bangladesh	The Philippines
Marketing mix expectations			
Product expectations	EProd	3.61 (0.54)	3.82 (0.64)
Price expectations	EPrice	3.27 (0.57)	3.42 (0.73)
Place expectations	EPlace	3.51 (0.85)	3.47 (0.92)
Promotion expectations	EPromo	3.96 (0.59)	4.19 (0.73)
Performance Expectations	PE	3.60 (0.68)	3.84 (0.75)
Expected Satisfaction	ES	3.75 (0.64)	3.76 (0.79)
Risk Perceptions	RP	2.76 (0.66)	2.87 (0.77)
Purchase Intention	PI	3.81 (0.74)	3.74 (0.84)

SD = Standard deviation.

In summary, the data suggests that while consumers in both countries have high purchase intentions for GR, there are slight differences in their expectations and perceptions. Consumers in the Philippines tend to have higher expectations for the product, price, promotion, and performance but also perceive slightly more risks compared to consumers in Bangladesh. Understanding these nuances can help tailor marketing strategies to better address consumers’ specific expectations and concerns in each region.

The standard deviations in Table 2 represent the variability or dispersion of responses around the mean for each country. The lower standard deviations in Bangladesh for different components of marketing mix expectations, perceptions, and purchase intentions indicate that responses are more closely clustered around the mean, reflecting greater agreement among respondents. In contrast, the higher standard deviations for several components in the Philippines suggest more variability in responses, indicating less consensus among respondents. Thus, overall, the data highlight that respondents in the Philippines tend to have more diverse perceptions and expectations than those in Bangladesh.

### 4.2. Influence of Marketing Mix Expectations on Performance Expectation, Expected Satisfaction, and Purchase Intention

#### 4.2.1. Verification of the Proposed Model and Hypotheses

A two-step approach to assess the proposed model’s relationships was employed. Structural equation modeling (SEM), a confirmatory method examining structured causal links,^74,75,76^ was used to analyze both measurement and structural models with SPSS AMOS, version 23. SEM’s consistency and efficiency in examining complex associations and hypotheses are well recognized.^77,78^ Before testing the research hypotheses, the measurement model’s goodness-of-fit was verified.^75^ The analysis began with the marketing expectation dimensions concerning the marketing mix, followed by the proposed research model’s examination and verification. Data are considered normally distributed when skewness is between ‐2 and +2, and kurtosis is between ‐7 and +7, as recommended.^76,79^ Based on these criteria, the data in our study meet the assumption of normality. While in AMOS, a significant Mardia coefficient may indicate non-normal data, this test is highly sensitive to sample size, with larger samples more likely to produce significant results, even for minor deviations from normality. Given our sample sizes for both countries, we instead applied a robust estimation method using the bootstrapping technique (with 5000 replicates). This approach helps mitigate issues with non-normal data and heteroscedasticity, providing more accurate parameter estimates and confidence intervals. In our analysis, we compared the bootstrapped standard errors with the default standard errors to evaluate any heteroscedasticity concerns. The consistency of the standard errors across both methods indicates that the assumption of homoscedasticity has been satisfied. Multicollinearity tests ensured no highly correlated variables. Model fitness tests included the comparative fit index (CFI), goodness-of-fit index (GFI), adjusted goodness-of-fit index (AGFI), Bentler – Bonett Normed Fit Index (NFI), chi-square value to the degree of freedom (chi-square/df), and root mean square error of approximation (RMSEA). To address the common method bias (CMB) in self-administered surveys, Harman’s single-factor test was performed and confirmed no CMB, with scores under 50% (BD = 37.14% and PHIL = 36.70%).^80^ Thus, no single factor accounted for most variances between items, indicating the absence of CMB.^51^

#### 4.2.2. Measurement Model of Marketing Mix Expectations

**Figure 2.**
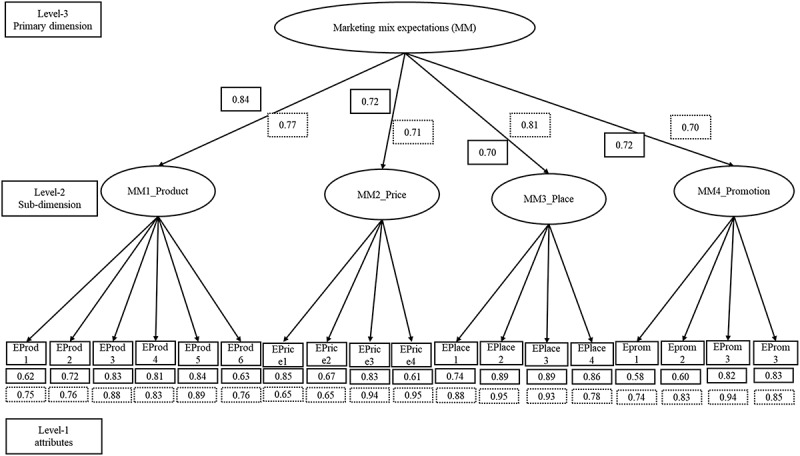
Measurement model of marketing mix expectations of golden rice.

At first, a three-level model structure was used to reflect the dimension-specific nature of the marketing mix expectations (see Figure 2). Eighteen items at level 1 represent the four dimensions of the marketing mix (product, price, place, promotion) at level 2, while consumers’ expectation of the marketing mix of GR at level 3 was, in turn, assessed through these four dimensions. Exploratory factor analysis (EFA) was conducted to identify core items shared in both subsamples and validate the marketing mix scale across Bangladesh and the Philippines, as well as the other latent variables used in the total measurement model. Before EFA, Bartlett’s test of sphericity (BTS) and Kaiser–Meyer–Olkin (KMO) analyses were performed, showing KMO values of 0.88 for Bangladesh and 0.89 for the Philippines, along with a significant BTS result (*p* = .000), indicating sufficient common variance for factor analysis. Principal Component Analysis with varimax rotation, eigenvalues greater than 1, and factor loadings greater than 0.60 were utilized in EFA.^81^ During item analysis, items with corrected item-total correlation coefficients below 0.40 were considered for deletion, and whether the removal of the item could significantly enhance the total reliability of the questionnaire was considered by using Cronbach’s alpha.^82^ The process was iterated until optimal results were achieved, as suggested by (Appendix B; Table B.1 and Table B.2) and resulted in 18 items after the removal of two items. The model-fit indices for the measurement model showed satisfactory values indicating that all values exceeded common acceptance levels^76,83,84^ (Appendix B; Table B.3). Subsequently, Confirmatory Factor Analysis (CFA) was conducted to assess validity and reliability, demonstrating adequate construct reliability, convergent validity, and discriminant validity. The χ^2^/df ratio, considered a good fit if below 3, was used, acknowledging χ^2’^s sensitivity to sample size.^85^ Additional fit indices were employed.^83^ Reliability analysis followed the recommendation of a reliability coefficient not less than 0.70.^86^ Results showed good reliability, with both Composite Reliability (CR) and Cronbach’s alpha values exceeding 0.70 for all constructs. Convergent validity was tested using Average Variance Extracted (AVE) and item loadings, with AVE values exceeding 0.50 for all constructs, indicating strong convergent validity^87^ in both countries. All factor loadings attained significance. Discriminant validity was assessed for both subsamples, and the square root of the AVE was greater than its correlation with other variables, indicating no discriminant validity issues.^84,87^ Based on these findings, the measurement model of consumers' marketing mix expectations of GR was deemed valid (see Appendix B; Table B.4 and Table B.5 for detailed CFA findings).

The straight-lined boxes indicate Bangladesh and the dotted-lined boxes indicate the Philippines

The values within the solid and dotted-line boxes represent the factor loadings for each item in Bangladesh and the Philippines, respectively, in the confirmatory factor analysis. A threshold of >0.60 for factor loadings was applied. Since all factor loadings exceeded this cutoff, we concluded that none of the factors were invalid. This figure provides a visual representation of the validation of the measurement model for marketing mix expectations, which was used as a second-order factor in the structural model of the study.

#### 4.2.3. Total Measurement Model

Initially, EFA was conducted to refine the scale for each construct, leading to the removal of the same four items, as shown in Appendix C (Tables C.1 and C.2) for Bangladesh and the Philippines, respectively. This was done following the same deletion criteria outlined in section 4.2.2. Next, we examined the full model. All item loadings exceeded 0.7, indicating strong internal reliability (see Table 3). A second-order factor was assessed using the constituent items of its lower-order factors to delineate their relationships.^88^ In SEM, this method computes second-order factors by incorporating multiple first-order factors. This approach was employed to construct the second-order variable (marketing mix expectations) and is common in literature.^65,66,89^ This approach has different characteristics. First, theoretically, the second-order constructs should be formed by the first-order constructs. Second, a moderate rather than a high level of correlation among the first-order constructs should be expected. Third, a low collinearity among the first-order constructs is expected. All these criteria were tested using marketing mix expectations as a second-order factor. To this end, MM1, MM2, MM3, and MM4 were calculated as first-order factors on the basis of the items under the product, price, place, and promotion dimensions. Cronbach’s alpha for all constructs was above 0.70, indicating adequate reliability. Composite reliability (CR) and average variance extracted (AVE) showed satisfactory convergent validity, with AVE values surpassing 0.50. Table 4 presents factor correlations and the square root of AVE, confirming discriminant validity as the square root of AVE was greater than the correlations between constructs. Detailed results, including satisfactory fit indices, are provided in Appendix C (Table C.3).

**Table 3.** Factor loadings and convergent validity results of the measurement model.

		Factor loadings		AVE		CR		Cronbach’s alpha	
Constructs	Items	BD	PHIL	BD	PHIL	BD	PHIL	BD	PHIL
Marketing Mix Expectations (MM)	Product	0.84	0.77	0.56	0.56	0.83	0.84	0.83	0.84
	Price	0.72	0.71						
	Place	0.70	0.81						
	Promotion	0.72	0.70						
Risk Perceptions (RP)	RP_1	0.75	0.83	0.59	0.67	0.85	0.89	0.85	0.89
	RP_2	0.88	0.91						
	RP_3	0.73	0.79						
	RP_4	0.70	0.72						
Performance Expectations (PE)	PE_1	0.78	0.86	0.69	0.81	0.90	0.95	0.89	0.94
	PE_2	0.92	0.93						
	PE_3	0.88	0.94						
	PE_4	0.73	0.87						
Expected Satisfaction (ES)	ES_1	0.82	0.94	0.73	0.90	0.91	0.97	0.91	0.97
	ES_2	0.82	0.98						
	ES_3	0.86	0.99						
	ES_4	0.92	0.88						
Purchase Intention (PI)	PI_1	0.63	0.94	0.64	0.88	0.87	0.97	0.88	0.97
	PI_2	0.66	0.90						
	PI_3	0.95	0.97						
	PI_4	0.91	0.93						

BD = Bangladesh; PHIL = The Philippines; AVE = Average Variance Extracted; CR = Composite Reliability.

**Table 4.** Factor correlations and discriminant validity of measurement model.

	Bangladesh					The Philippines				
	PI	PE	ES	RP	MM	PI	PE	ES	RP	MM
PI	**[0.80]**					**[0.94]**				
PE	0.37	**[0.83]**				0.28	**[0.90]**			
ES	0.58	0.51	**[0.85]**			0.41	0.48	**[0.95]**		
RP	−0.35	−0.19	−0.25	**[0.77]**		−0.30	−0.25	−0.33	**[0.82]**	
MM	0.43	0.48	0.49	−0.22	**[0.75]**	0.21	0.42	0.30	−0.07	**[0.75]**

Diagonal elements (bold) show the square root of average variance extracted (AVE). Off-diagonal elements show the shared variance.

PI = Purchase intention; PE = Performance expectations; ES = Expected satisfaction; RP = Risk perceptions; MM = Marketing mix expectations.

#### 4.2.4. Structural Model

**Figure 3.**
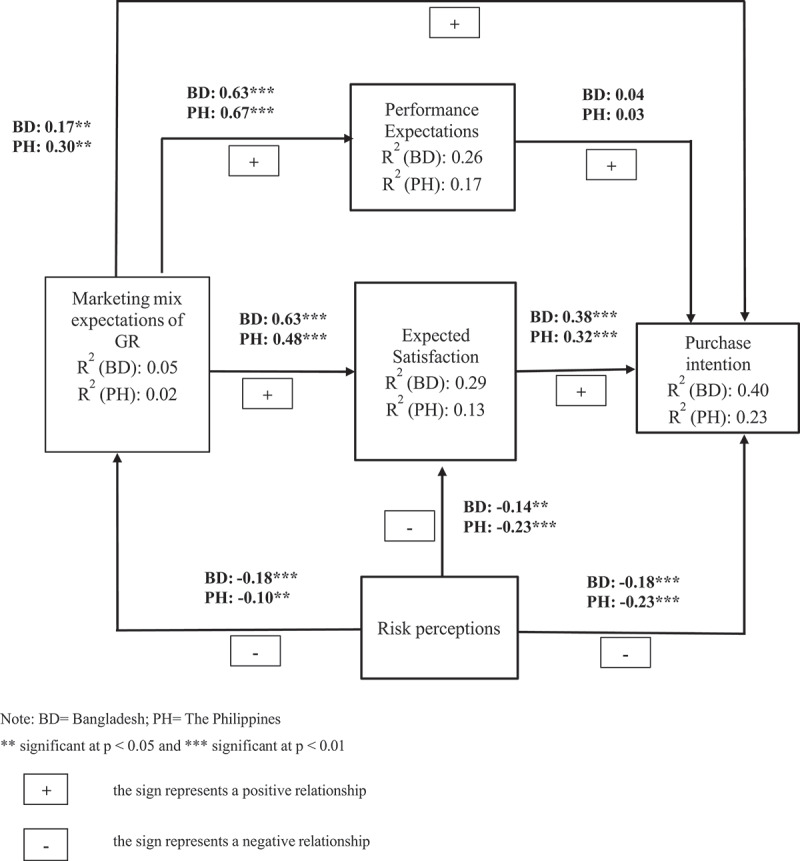
Standardized structural relationship between latent variables related to golden rice in for Bangladesh and the Philippines.

The second step of structural equation modeling is the evaluation of the structural model, which is used to analyze the relationships between the latent variables. Our structural model had five latent variables (Marketing mix expectations, performance expectations, expected satisfaction, risk perceptions, and purchase intention). Figure 2 provided an intuitive explanation of the consumers’ marketing mix expectations, based on the assessment of the four sub-dimensions, namely product, price, place, and promotion, and all achieved by evaluating a range of specific product attributes. The goodness of fit of the model must be assessed before the parameters of the model are estimated. The goodness of fit measures showed satisfactory values including (χ^2^/df = 2.03; GFI = 0.92; TLI = 0.96; CFI = 0.97; NFI = 0.94; RMSEA = 0.05; RMR = 0.05) for Bangladesh and (χ^2^/df = 2.05; GFI = 0.92; TLI = 0.97; CFI = 0.98; NFI = 0.95; RMSEA = 0.05; RMR = 0.07) (see Appendix D, Table D.1). Given the model’s excellent fit, the hypotheses for the data in Bangladesh and the Philippines were next evaluated using the predicted path coefficients of the structural model. Path coefficients and the R^2^ were used jointly to evaluate the model. The path coefficients show the strength of the correlations between the dependent and independent variables, while the R^2^ values represent the percentage of variance explained by the independent variables. Figure 3 shows the structural path relationships and corresponding coefficients for each country analysis. All the hypotheses were strongly supported in both Bangladesh and the Philippines, except hypothesis H4 (see Appendix D, Table D.2).

**significant at *p* < .05 and ***significant at *p* < .01.

In both countries, consumers’ expectations of the marketing mix had a significant positive influence on performance expectations of GR, expected satisfaction at the pre-purchase stage, and purchase intention, confirming H1-H3. However, H4 was rejected in both countries, indicating a lack of significant effect of consumers’ performance expectations on GR purchase intentions. The path coefficient for H5 was again positive and significant for both countries. As for consumers’ risk perceptions, the expected negative influence from GR marketing mix expectations (H6) was verified for both countries. Finally, we lend support for the negative influence of GR risk perceptions on their expected satisfaction at the pre-purchase stage (H7) and purchase intentions (H8).

In summary, a consistent trend is observed across both countries in explaining consumers’ purchase intentions toward GR. However, country discrepancies arise in the relationships between consumer risk perceptions on the one hand and marketing mix expectations and expected satisfaction on the other. Although consumers’ marketing mix expectations of GR were negatively associated with risk perceptions in both Bangladesh and the Philippines, the effect is more pronounced in the former. Conversely, the link between risk perceptions and expected satisfaction is stronger in the Philippines.

### 4.3. Multiple Linear Regression Analysis

In the final step, we delve deeper into the effects of specific components of the marketing mix, i.e., product, price, place, and promotion, on the expected performance, satisfaction, and purchase intention of GR. To this end, a series of multiple linear regressions was conducted (Table 5). The product-related expectations positively influenced every outcome variable across all countries. Consumers’ GR price expectations had a significant, albeit small, negative impact on purchase intention, but only in Bangladesh. In contrast, consumer expectations of the place (distribution) positively influenced all outcomes in the Philippines, except for purchase intention. However, the coefficient values were on the small side, indicating a limited effect. Lastly, consumers’ expectations of GR promotion positively influenced all three outcome variables across all countries.

**Table 5.** Effects of consumer expectations of the four marketing mix dimensions on performance expectations, expected satisfaction, and purchase intentions.

	Performance expectations				Expected satisfaction				Purchase intention			
Marketing expectations	Bangladesh		The Philippines		Bangladesh		The Philippines		Bangladesh		The Philippines	
	Stand. Coeff. (β)	t	Stand. Coeff. (β)	t	Stand. Coeff. (β)	t	Stand. Coeff. (β)	t	Stand. Coeff. (β)	t	Stand. Coeff.(β)	t
Product	0.37***	7.742	0.27***	5.014	0.28***	5.803	0.19***	3.410	0.26***	5.467	0.23***	3.934
Price	0.01	0.135	0.07	1.427	0.04	0.898	0.05	0.888	−0.09**	−2.11	−0.03	−0.515
Place	0.10**	2.161	0.19***	3.411	0.14***	2.892	0.10*	1.827	0.12***	2.617	−0.003	−0.048
Promotion	0.25***	5.132	0.18***	3.569	0.26***	5.385	0.28***	5.449	0.35***	7.221	0.24***	4.450
	R^2^ = 0.328, Adj-R^2^ = 0.322,		R^2^ = 0.285, Adj-R^2^ = 0.277,		R^2^ = 0.299, Adj-R^2^ = 0.292,		R^2^ = 0.224, Adj-R^2^ = 0.215,		R^2^ = 0.312, Adj-R^2^ = 0.305,		R^2^ = 0.146, Adj-R^2^ = 0.136,	
	F = 47.206***		F = 34.737***		F = 41.206***		F = 25.168***		F = 43.756***		F = 14.938***	

*p < .05. **p < .01. ***p < .001.

Stand. Coff = Standard coefficient.

## 5. Discussion

GR is one of the most advanced applications of GM biofortification, with the Philippines being the first country to progress toward commercial propagation and Bangladesh moving toward the last stages of legalization. However, understanding consumer behavior, which is vital for the successful marketing of new products, requires an analysis of theoretical frameworks.^90^ Given that positive expectations of consumers about product characteristics predict purchase behavior,^91,92^ this study partially relied on ECT.^48^ This theory successfully explained linkages between expectation, satisfaction, and post-purchase behaviors across diverse industries, including internet services, online banking, restaurants, and e-learning.^61,64,93^ It extends beyond marketing to sociology, information technology, and tourism.^94–96^ The original ECT process involves pre- and post-stage evaluation of consumer behavior. As consumers develop expectations and perceptions, respectively, before and after experiencing the product, a comparison of both determines the level of confirmation. When expectations are not met, it results in dissatisfaction and reluctance to (re)purchase.

Despite its widespread use in marketing, ECT has seen limited application in predicting consumer expectations for food products before the market launch.^30^ Researchers also suggested adding constructs to further improve its predictive power in explaining consumer purchasing behavior.^97–99^ This study extends this model by incorporating risk perceptions to better understand consumer purchase intentions for GM biofortified foods. In the context of nutrition security, perceived risk is crucial, influencing consumer behavior toward emerging technologies like GM.^31,70,100^ As GR is not yet commercially available, consumers obviously lack direct experience with it. Therefore, the original ECT variables have been adapted to include five constructs: expectations of the marketing mix components, performance expectations, expected satisfaction, risk perceptions, and purchase intention. Given the current status of GR, we excluded the construct “confirmation,” which requires product experience.

The modified ECT model included consumer expectations toward the four key components of the marketing mix, also known as the 4Ps of marketing, i.e., product, price, place, and promotion, a fundamental framework in strategic marketing management. First, consumers evaluate food based on product characteristics such as safety, packaging, functionality, labeling, and brand.^101^ Previous biofortification research typically looked at attributes like taste, texture, appearance, health perceptions, and nutritional content.^23,102–104^ Second, price is a key driver of consumer preferences, shaping perceptions of quality, value, and affordability.^105,106^ Fair pricing is expected to enhance satisfaction and post-consumption experience.^61^ Third, convenient accessibility through appropriate distribution channels can boost satisfaction and purchase intentions.^107^ Direct availability increases future purchases, emphasizing its role in the marketing mix.^60^ Fourth, promotional efforts have been shown to influence consumer behavior,^108^ especially when the communication is tailored to the product and audience.^109^ Effective promotion enhances expectations, leading to increased satisfaction and purchase motivation.^108^ Therefore, marketing mix expectations, as a whole, are assumed to notably influence performance, with higher expectations resulting in higher performance scores and increased satisfaction.^61,110^

The findings of this study suggest that price expectations do not affect expected satisfaction, performance, or purchase intention in the Philippines, which is also true for Bangladesh, except for a slightly significant negative impact on purchase intention. Other marketing mix elements have a much greater influence on these outcomes, though their effects vary between countries. For example, while higher expectations for product, place, and promotion lead to higher expected satisfaction with GR in both countries, product expectations have a larger (Bangladesh) and smaller (the Philippines) influence on expected satisfaction than promotion. Additionally, expectations regarding the place have a larger influence on expected satisfaction in Bangladesh compared to the Philippines. While consumers from both countries have the highest expectations regarding the product and promotion characteristics of GR, followed by place, the former two do not affect expected satisfaction equally in both countries, highlighting different influences of marketing mix expectations on the expected satisfaction with GR. These findings have echoed in the previous literature.^111,112^

Furthermore, the study found that in Bangladesh, all elements of marketing mix expectations significantly influence consumers’ purchase intention for GR, while in the Philippines, only product and promotion expectations appear to be significant. The importance of the latter two P’s as significant determinants of purchase intention in both countries is further strengthened by their relatively higher mean score by the respondents. This finding aligns with prior research, where the effect of different P’s of the marketing mix has been examined on consumers’ purchase intentions for innovative foods and new technologies across several products and countries.^34,60,113,114^ Literature shows that when buying novel food products, consumers often rely on objective features like nutritional value, health benefits, and low risks.^33,115^ Positive expectations toward these aspects of the product increase their intention to purchase, as confirmed by the current study’s findings.

Performance expectation refers to consumers’ subjective anticipation of a product’s effectiveness, developed through direct or indirect engagement over time.^116^ For technological innovations, consumers’ beliefs about how the technology aligns with their goals are crucial for generating positive performance expectations,^117^ which are assumed to positively impact their behavioral intentions toward the technology. Hence, it is reasonable to infer that consumers will be inclined to purchase GR when they anticipate obtaining benefits from its consumption. Biofortification can enhance nutrient intake levels, as shown in efficacy and simulation studies,^23,118,119^ which may foster positive expected performance. In the case of GR as a GM biofortified crop, the nutrition benefits are expected to improve consumer perceptions.^120,121^

Performance expectations did not influence the purchase intention for both country samples. While consumers have high expectations about the performance of GR in both countries and especially in the Philippines, consumers’ purchase intention toward GR does not seem to depend on these expectations. However, this might change when consumers actually experience GR, as this consumption experience is expected to influence performance expectations.

Consumer satisfaction is an affective state that represents a consumer’s reaction after fulfilling initial product expectations.^68^ The more consumers feel that their expectations for the GR would be fulfilled, the more satisfied they are expected to be. Effective marketing efforts that emphasize the positive attributes of GR can play a crucial role in shaping consumers’ expectations and contributing to their expected satisfaction and subsequent purchase intention. The influence of consumers’ expectations on satisfaction on the one hand and of satisfaction on purchase intention on the other is well established^64^ and has been confirmed in food studies targeting specific settings such as restaurants^122^ and shopping apps,^123^ as well as specific products, such as vegetables.^124^

Indeed, also for this study, it was found that expected satisfaction has a substantial influence on consumers’ purchase intention of GR in both countries, consistent with prior research.^30,125^ This underscores the importance of consumer satisfaction as a reliable indicator of purchase intention, aligning with previous findings.^126^

Previous research has often explored the link between risk perceptions and consumers’ purchase intentions for GM crops in both developed^37^ and developing countries.^35^ This study identified a negative relationship between risk perceptions, marketing mix expectations, expected satisfaction, and purchase intention, which had not been explored in the previous research. Nevertheless, numerous studies on GM (biofortified) foods have demonstrated that consumers’ risk perceptions significantly influence their purchase intentions.^35,127,128^ This study also provides valuable insights into how perceived risks can affect marketing mix expectations and expected satisfaction. The findings show that in Bangladesh, consumers’ risk perceptions strongly affect their marketing mix expectations and purchase intentions, whereas, in the Philippines, the effect is more prominent on expected satisfaction and purchase intention. This indicates that risk perceptions may influence not only purchase intentions directly but also indirectly through their influence on marketing mix expectations and expected satisfaction. Previous consumer research on novel foods^129,130^ consistently showed a significant negative correlation between perceived risks and satisfaction. It is evident from the findings that when consumers associate high risks with, e.g., the potential side effects of GR consumption, social ethics, and human values, and the authenticity of the information on nutritional benefits, this can affect their marketing mix expectations^33^ and their purchase intentions.^70,131^ Being informed can remarkably reduce consumers’ fear and perception of risk associated with a product.^128,132^ Knowledge helps consumers to understand the product better, preventing them from prematurely rejecting potential benefits.^133^

Theoretically, this study has shown that our partial ECT-based model can be applied to foods that have not yet found entrance on the market. While the majority of ECT research looked at established products, our study constructed and validated a relevant part of this model for evaluating new, controversial products before launch. Moreover, through operationalizing marketing mix expectations as a second-order factor, and integrating risk perceptions into our model, we further enhance our understanding of determinants of consumers’ expectations, expected satisfaction, and purchase intention. The extended model can be used to guide research on post-acceptance behavior.

This study has several limitations. Firstly, the utilization of an online survey may not be optimal for addressing the nuances of the target audience for GR. Future studies should focus on rural areas to investigate their expectations and perceptions of GM biofortified foods like GR. They may show differences compared to urban areas in terms of their expectations and perceptions about GM biofortified foods as they usually have less awareness about the technology as well as deep-rooted cultural practices related to agriculture and food consumption. Therefore, future research should also prioritize addressing rural areas’ unique challenges by considering local agricultural practices and dietary habits, developing region-specific educational programs and informational campaigns, and analyzing and comparing market value chains in rural (and urban) contexts. Secondly, this investigation was conducted during the pre-purchase stage of GR in both countries, predominantly involving a younger sample. To mitigate these limitations, future research endeavors should encompass respondents from in-person surveys and assess potential variations in the impact of each of the four offer elements on purchasing decisions across diverse consumer segments. In addition, conducting longitudinal studies may allow us to capture the variation of the influence of these four elements over time. Future research could also evaluate the original ECT model based on post-purchase surveys once GR is available, as well as other GM biofortified food crops, allowing consumers to confirm their expectations through real consumption experiences. Recognizing the imperative role of optimizing the marketing mix components in the market development of GM biofortified food crops, similar research could be extended in different developing regions using advanced experimental designs.

## 6. Conclusion

Through emphasizing the importance of consumer “expectations and perceptions” in predicting the market potential of GR in the target countries, Bangladesh and the Philippines, this study offers critical insights for various stakeholders, including developers, producers, marketers, and health planners. First of all, marketing mix expectations exert a direct influence on the purchase intentions of GR, as well as on the expected performance and satisfaction. When looking at the specific marketing mix dimensions, expectations related to the product characteristics and promotion of GR drive consumers’ interest in both countries. Product-related factors such as taste, durability, and overall quality need to be prioritized by product developers to meet consumer expectations. Promotional efforts through transparent and informative communication about GR will also play an important role. A tailored information campaign to inform consumers about the product characteristics of GR, emphasizing the nutritional benefits it offers, is expected to contribute to a successful commercialization. Thereby, marketers could establish promotional strategies such as product labeling or packaging to clearly communicate the distinct characteristics of GR. Other marketing mix dimensions, such as place and price, were only driving consumer purchase intentions in Bangladesh, calling for country-specific marketing strategies to accommodate marketing mix sensitivities.

Finally, this study found that more negative risk perceptions about GR reduce consumers’ marketing mix expectations, expected satisfaction, and purchase intention. Even if marketers heavily invest in GR marketing, it will be crucial to increase GR’s trustworthiness and counter any potential negative effects of misinformation that are likely to occur before or after commercialization. In spite of this, our study illustrates that a well-designed marketing strategy for GR could further enhance its success in its expected target markets in the Philippines and Bangladesh.

## Supplementary Material

Appendix C_revised clean.docx

Appendix A_revised clean.docx

Appendix D_revised clean.docx

Appendix B_revised clean.docx
