# Supplementary material for: Golden opportunities? How marketing expectations drive purchase intentions of golden rice in Bangladesh and the Philippines
Source: GM Crops Food. 2024 Nov 18;15(1):316–35. doi: 10.1080/21645698.2024.2418161 (PMC11581164; doi:10.1080/21645698.2024.2418161)
Supplement: Appendix C_revised clean.docx [file KGMC_A_2418161_SM6860.docx]

**Appendix C.** Total measurement model

**Table C.1:** Exploratory factor analysis for the overall measurement model in Bangladesh

| Factor name | Items | Factor 1 | Factor 2 | Factor 3 | Factor 4 | Factor 5 |
| --- | --- | --- | --- | --- | --- | --- |
| Purchase intention | PI_1 |  |  | 0.71 |  |  |
|  | PI_2 |  |  | 0.72 |  |  |
|  | PI_3 |  |  | 0.84 |  |  |
|  | PI_4 |  |  | 0.84 |  |  |
| Performance Expectations | PE_1 |  | 0.83 |  |  |  |
|  | PE_2 |  | 0.85 |  |  |  |
|  | PE_3 |  | 0.84 |  |  |  |
|  | PE_4 |  | 0.79 |  |  |  |
| Expected Satisfaction | ES_1 | 0.77 |  |  |  |  |
|  | ES_2 | 0.80 |  |  |  |  |
|  | ES_3 | 0.82 |  |  |  |  |
|  | ES_4 | 0.85 |  |  |  |  |
| Risk Perceptions | RP_1 |  |  |  | 0.82 |  |
|  | RP_2 |  |  |  | 0.86 |  |
|  | RP_3 |  |  |  | 0.82 |  |
|  | RP_4 |  |  |  | 0.76 |  |
| Marketing mix  Expectations | MM_1 |  |  |  |  | 0.77 |
|  | MM_2 |  |  |  |  | 0.84 |
|  | MM_3 |  |  |  |  | 0.75 |
|  | MM_4 |  |  |  |  | 0.73 |

Note: Extraction Method: Principal Component Analysis. Rotation Method: Varimax with Kaiser Normalization. Rotation converged in 6 iterations

KMO: 0.88; Bartlett’s Test of Sphericity: Approximate Chi-square (4969.67; Sig. 0.000)

Total Variance explained: 73.72%

**Table C.2:** Exploratory factor analysis for overall measurement model in the Philippines

| Factor name | Items | Factor 1 | Factor 2 | Factor 3 | Factor 4 | Factor 5 |
| --- | --- | --- | --- | --- | --- | --- |
| Purchase intention | PI_1 |  | 0.92 |  |  |  |
|  | PI_2 |  | 0.91 |  |  |  |
|  | PI_3 |  | 0.93 |  |  |  |
|  | PI_4 |  | 0.90 |  |  |  |
| Performance Expectations | PE_1 |  |  | 0.87 |  |  |
|  | PE_2 |  |  | 0.90 |  |  |
|  | PE_3 |  |  | 0.89 |  |  |
|  | PE_4 |  |  | 0.87 |  |  |
| Expected Satisfaction | ES_1 | 0.91 |  |  |  |  |
|  | ES_2 | 0.92 |  |  |  |  |
|  | ES_3 | 0.93 |  |  |  |  |
|  | ES_4 | 0.89 |  |  |  |  |
| Risk Perceptions | RP_1 |  |  |  | 0.84 |  |
|  | RP_2 |  |  |  | 0.89 |  |
|  | RP_3 |  |  |  | 0.85 |  |
|  | RP_4 |  |  |  | 0.81 |  |
| Marketing mix  Expectations | MM_1 |  |  |  |  | 0.79 |
|  | MM_2 |  |  |  |  | 0.82 |
|  | MM_3 |  |  |  |  | 0.82 |
|  | MM_4 |  |  |  |  | 0.77 |

Note: Extraction Method: Principal Component Analysis. Rotation Method: Varimax with Kaiser Normalization. Rotation converged in 6 iterations

KMO: 0.88; Bartlett’s Test of Sphericity: Approximate Chi-square (7135.22; Sig. 0.000)

Total Variance explained: 82.58%

Total deleted items (4): Expected Satisfaction (2); Risk perception (1); Purchase intention (1)

**Decision criteria for deletion of items:** Corrected item-total correlation coefficients below 0.40 were considered for deletion,and whether the removal of the item could significantly enhance the total reliability of the questionnaire was considered by using Cronbach’s alpha (Kuo et al., 2009)

The Confirmatory factor analysis (CFA) was then employed to examine connections between latent and observable variables. The goodness of fit measures showed satisfactory values (χ^2^/df = 1.80; GFI = 0.93; TLI = 0.97; CFI = 0.97; NFI = 0.94; RMSEA = 0.04; RMR = 0.03) for Bangladesh and (χ^2^/df = 1.64; GFI = 0.93; TLI = 0.98; CFI = 0.98; NFI = 0.96; RMSEA = 0.04; RMR = 0.02) (Table C.3). The common fit indices demonstrated that all model fit indices met proposed cutoffs, indicating an overall satisfactory model fit.

A second-order factor was assessed using the constituent items of its lower-order factors to delineate their relationships (Edwards, 2001). In SEM, this method computes second-order factors by incorporating multiple first-order factors. This approach was employed to construct the second-order variable (marketing mix expectations), and is common in literature (Lin et al., 2012; Qazi et al., 2017; Wang et al., 2019). This approach has different characteristics. First, theoretically, the second-order constructs should be formed by the first-order constructs. Second, a moderate rather than a high level of correlation among the first-order constructs should be expected. Third, a low collinearity among the first-order constructs is expected. All these criteria were tested using marketing mix expectations as a second-order factor. To this end, MM1, MM2, MM3, and MM4 were calculated as first-order factors on the basis of the items under the product, price, place, and promotion dimensions.

**Table C.3:** The fit indices and analysis results of the overall measurement model

| Fit indices | Recommended value | Result | |
| --- | --- | --- | --- |
|  |  | Bangladesh | The Philippines |
| χ^2^/df | <3.00 | 1.80 | 1.64 |
| GFI (goodness of fit index) | >0.90 | 0.93 | 0.93 |
| RMSEA (root mean square error of approximation) | <0.08 | 0.04 | 0.04 |
| RMR (root mean square residual) | <0.08 | 0.03 | 0.02 |
| NFI (normed fit index) | >0.90 | 0.94 | 0.96 |
| TLI (tucker-lewis index) | >0.90 | 0.97 | 0.98 |
| CFI (comparative fit index) | >0.90 | 0.97 | 0.98 |

**References**

Edwards, J. R. (2001). Multidimensional constructs in organizational behavior research: An integrative analytical framework. *Organizational research methods*, *4*(2), 144-192.

Kuo, Y.-F., Wu, C.-M., & Deng, W.-J. (2009). The relationships among service quality, perceived value, customer satisfaction, and post-purchase intention in mobile value-added services. *Computers in Human Behavior*, *25*(4), 887-896.

Lin, T.-C., Wu, S., Hsu, J. S.-C., & Chou, Y.-C. (2012). The integration of value-based adoption and expectation–confirmation models: An example of IPTV continuance intention. *Decision Support Systems*, *54*(1), 63-75. <https://doi.org/10.1016/j.dss.2012.04.004>

Qazi, A., Tamjidyamcholo, A., Raj, R. G., Hardaker, G., & Standing, C. (2017). Assessing consumers' satisfaction and expectations through online opinions: Expectation and disconfirmation approach. *Computers in Human Behavior*, *75*, 450-460. <https://doi.org/10.1016/j.chb.2017.05.025>

Wang, M. Y., Zhang, P. Z., Zhou, C. Y., & Lai, N. Y. (2019). Effect of Emotion, Expectation, and Privacy on Purchase Intention in WeChat Health Product Consumption: The Mediating Role of Trust. *Int J Environ Res Public Health*, *16*(20). <https://doi.org/10.3390/ijerph16203861>
