# Supplementary material for: Golden opportunities? How marketing expectations drive purchase intentions of golden rice in Bangladesh and the Philippines
Source: GM Crops Food. 2024 Nov 18;15(1):316–35. doi: 10.1080/21645698.2024.2418161 (PMC11581164; doi:10.1080/21645698.2024.2418161)
Supplement: Appendix A_revised clean.docx [file KGMC_A_2418161_SM6859.docx]

**Appendix A.**

**Table A.1:** Summary of specific items of the constructs

| Variables/  Abbreviation | | Descriptives | Mean | | | SD | | | |
| --- | --- | --- | --- | --- | --- | --- | --- | --- | --- |
|  |  |  | BD | PHIL | | BD | | PHIL | |
| Marketing Mix expectations | Expectations towards product (GR) (EProd) | **EProd1:** Tasty to eat | 3.39 | 3.66 | | 0.631 | | 0.736 | |
|  |  | **EProd2:** High Quality | 3.86 | 3.93 | | 0.755 | | 0.794 | |
|  |  | **EProd3:** Reliable | 3.69 | 3.88 | | 0.715 | | 0.756 | |
|  |  | **EProd4:** Durable | 3.54 | 3.74 | | 0.659 | | 0.742 | |
|  |  | **EProd5:** Dependable | 3.61 | 3.74 | | 0.689 | | 0.742 | |
|  |  | **EProd6:** Informative packaging | 3.56 | 3.98 | | 0.657 | | 0.764 | |
|  | Expectation toward Price (EPrice) | **EPrice1:** Lower than what I prefer | 3.20 | 3.29 | | 0.689 | | 0.872 | |
|  |  | **EPrice2:** Lower than average market price | 3.19 | 3.30 | | 0.751 | | 0.897 | |
|  |  | **EPrice3:** Reasonable | 3.32 | 3.58 | | 0.671 | | 0.807 | |
|  |  | **EPrice4:** Negotiable | 3.38 | 3.53 | | 0.712 | | 0.793 | |
|  | Expectation toward Place  (EPlace) | **EPlace1:** Readily available to purchase | 3.41 | 3.46 | | 0.957 | | 0.958 | |
|  |  | **EPlace2:** Easily accessible in the outlet stores | 3.66 | 3.43 | | 0.942 | | 1.036 | |
|  |  | **EPlace3:** Convenient to get to any location without any supply chain disruption | 3.39 | 3.34 | | 1.009 | | 1.047 | |
|  |  | **EPlace4:** Information on where I can source Golden Rice is readily available | 3.59 | 3.64 | | 0.948 | | 0.961 | |
|  | Expectation toward Promotion  (EPromo) | **EProm1:** The salesperson should be capable of giving enough information | 3.83 | 4.23 | | 0.716 | | 0.851 | |
|  |  | **EProm2:** Various promotion campaigns for Golden Rice should be offered (e.g., additional free products for purchasing a certain amount) | 3.84 | 4.10 | | 0.780 | | 0.822 | |
|  |  | **EProm3:** Advertisements for Golden rice should be available frequently | 4.10 | 4.20 | | 0.766 | | 0.822 | |
|  |  | **EProm4:** Public relations materials should be readily available for reference (e.g., newsletters, annual reports, and bulletins) | 4.05 | | 4.21 | 0.719 | 0.793 | | |
| Risk Perception (RP) | | **RP1:** Golden rice can have dangerous side effects on human beings such as allergies, toxicity | 2.76 | | 2.77 | 0.751 | 0.871 | | |
|  |  | **RP2:** There are ethical risks from Golden rice | 2.65 | | 2.83 | 0.795 | 0.900 | | |
|  |  | **RP3:** The vested interest groups control the risks of Golden rice | 2.86 | | 2.95 | 0.812 | 0.850 | | |
|  |  | **RP4:** If I were buying, I would worry about the reliability of the information provided by the product | 2.79 | | 2.93 | 0.827 | 0.941 | | |
| Performance Expectation (PE) | | **PE1:** The normal cooking method will not destroy the nutrient content of Golden Rice | 3.48 | | 3.83 | 0.737 | 0.816 | | |
|  |  | **PE2:** Consumption of Golden Rice will allow me to reach the required vitamin A intake level | 3.71 | | 3.89 | 0.794 | 0.786 | | |
|  |  | **PE3:** Consumption of Golden Rice will assist me to reduce night blindness-related problems | 3.77 | | 3.85 | 0.818 | 0.776 | | |
|  |  | **PE4:** Storage of Golden Rice for a longer period will keep the stability of nutrient content in the grain | 3.46 | | 3.78 | 0.789 | 0.855 | | |
| Expected Satisfaction (ES) | | **ES1:** I would feel satisfied if Golden rice becomes available in the market | 3.77 | | 3.82 | 0.744 | 0.833 | | |
|  |  | **ES2:** I would feel satisfied with my decision of buying Golden Rice | 3.73 | | 3.77 | 0.762 | 0.804 | | |
|  |  | **ES3:** I would feel satisfied with my decision of consuming Golden Rice | 3.75 | | 3.77 | 0.729 | 0.804 | | |
|  |  | **ES4:** Golden rice would not give me better satisfaction in comparison with other varieties of rice I consumed before | 3.75 | | 3.68 | 0.635 | 0.843 | | |
| Purchase Intention (PI) | | **PI1:** I would buy Golden Rice if it becomes available in the market | 3.80 | | 3.71 | 0.845 | 0.863 | | |
|  |  | **PI2:** I would consider buying Golden rice from a store if the quality and price are similar to other varieties | 3.94 | 3.75 | | 0.771 | | | 0.872 |
|  |  | **PI3:** I am willing to buy Golden rice | 3.75 | 3.74 | | 0.886 | | | 0.887 |
|  |  | **PI4:** I am willing to buy and willing to consume it | 3.74 | 3.76 | | 0.947 | | | 0.907 |

**Note:** BD= Bangladesh; PHIL= The Philippines

Total deleted items (6): Expectations towards product (GR) (Item 1_Visually appealing); Expectation toward Price ( Item 3_Expensive); Expected Satisfaction (2); Risk perception (1); Purchase intention (1)

We used corrected item-total correlation coefficients below 0.40 as a criterion for deletion, and whether the removal of the item could significantly enhance the total reliability of the questionnaire was considered by using Cronbach’s alpha (Kuo et al., 2009)

Kuo, Y.-F., Wu, C.-M., & Deng, W.-J. (2009). The relationships among service quality, perceived value, customer satisfaction, and post-purchase intention in mobile value-added services. *Computers in Human Behavior*, *25*(4), 887-896.
