# Supplementary material for: Golden opportunities? How marketing expectations drive purchase intentions of golden rice in Bangladesh and the Philippines
Source: GM Crops Food. 2024 Nov 18;15(1):316–35. doi: 10.1080/21645698.2024.2418161 (PMC11581164; doi:10.1080/21645698.2024.2418161)
Supplement: Appendix D_revised clean.docx [file KGMC_A_2418161_SM6858.docx]

**Appendix D.**

**Table D.1:** The fit indices and analysis results of the structural model

| Fit indices | Recommended value | Result | |
| --- | --- | --- | --- |
|  |  | Bangladesh | The Philippines |
| χ^2^/df | <3.00 | 2.03 | 2.05 |
| GFI (goodness of fit index) | >0.90 | 0.92 | 0.92 |
| RMSEA (root mean square error of approximation) | <0.08 | 0.05 | 0.05 |
| RMR (root mean square residual) | <0.08 | 0.05 | 0.07 |
| NFI (normed fit index) | >0.90 | 0.94 | 0.95 |
| TLI (tucker-lewis index) | >0.90 | 0.96 | 0.97 |
| CFI (comparative fit index) | >0.90 | 0.97 | 0.98 |

**Table D.2:** Summary of the hypothesis and diagnostic tests for Bangladesh and the Philippines

| Hypothesis | Hypothesized paths | | |  | Standardized regression coefficient | Critical Ratio  (C.R.) | Significance | Decision |
| --- | --- | --- | --- | --- | --- | --- | --- | --- |
| H1 | MM | 🡺 | PE | BD | 0.63*** | 8.240 | Significant | Accepted |
|  |  |  |  | PH | 0.67*** | 6.697 | Significant | Accepted |
| H2 | MM | 🡺 | ES | BD | 0.63*** | 8.129 | Significant | Accepted |
|  |  |  |  | PH | 0.48*** | 4.626 | Significant | Accepted |
| H3 | MM | 🡺 | PI | BD | 0.17** | 2.318 | Significant | Accepted |
|  |  |  |  | PD | 0.30** | 2.567 | Significant | Accepted |
| H4 | PE | 🡺 | PI | BD | 0.04 | 0.838 | Non-significant | Rejected |
|  |  |  |  | PH | 0.03 | .488 | Non-significant | Rejected |
| H5 | ES | 🡺 | PI | BD | 0.38*** | 6.911 | Significant | Accepted |
|  |  |  |  | PH | 0.32*** | 5.887 | Significant | Accepted |
| H6 | RP | 🡺 | MM | BD | -0.18*** | -3.803 | Significant | Accepted |
|  |  |  |  | PH | -0.10** | -2.518 | Significant | Accepted |
| H7 | RP | 🡺 | ES | BD | -0.14** | -2.585 | Significant | Accepted |
|  |  |  |  | PH | -0.23*** | -3.623 | Significant | Accepted |
| H8 | RP | 🡺 | PI | BD | -0.18*** | -4.029 | Significant | Accepted |
|  |  |  |  | PH | -0.23*** | -3.601 | Significant | Accepted |

Note: ** significant at p < 0.05 and *** significant at p < 0.01; PH = The Philippines, BD = Bangladesh, CR = composite reliability

PI= Purchase Intention; PE= Performance Expectations; ES= Expected Satisfaction; RP= Risk Perceptions; MM= Marketing Mix Expectations
