# Supplementary material for: Golden opportunities? How marketing expectations drive purchase intentions of golden rice in Bangladesh and the Philippines
Source: GM Crops Food. 2024 Nov 18;15(1):316–35. doi: 10.1080/21645698.2024.2418161 (PMC11581164; doi:10.1080/21645698.2024.2418161)
Supplement: Appendix B_revised clean.docx [file KGMC_A_2418161_SM6857.docx]

**Appendix B:** Measurement model of the marketing mix expectations

**Table B.1:** Expectations towards four dimensions of marketing mix by using Exploratory

Factor Analysis in Bangladesh of 18 items

| Factor name | Items | Factor 1 | Factor 2 | Factor 3 | Factor 4 |
| --- | --- | --- | --- | --- | --- |
| Product | Prod1 | 0.69 |  |  |  |
|  | Prod2 | 0.75 |  |  |  |
|  | Prod3 | 0.83 |  |  |  |
|  | Prod4 | 0.83 |  |  |  |
|  | Prod5 | 0.79 |  |  |  |
|  | Prod6 | 0.64 |  |  |  |
| Price | Price1 |  |  | 0.86 |  |
|  | Price2 |  |  | 0.77 |  |
|  | Price3 |  |  | 0.83 |  |
|  | Price4 |  |  | 0.70 |  |
| Place | Place1 |  | 0.78 |  |  |
|  | Place2 |  | 0.90 |  |  |
|  | Place3 |  | 0.89 |  |  |
|  | Place4 |  | 0.85 |  |  |
| Promotion | Prom1 |  |  |  | 0.63 |
|  | Prom2 |  |  |  | 0.79 |
|  | Prom3 |  |  |  | 0.77 |
|  | Prom4 |  |  |  | 0.80 |

Note: Extraction Method: Principal Component Analysis. Rotation Method: Varimax with Kaiser Normalization. Rotation converged in 5 iterations.

KMO: 0.88; Bartlett's Test of Sphericity: Approximate Chi-square (3755.70; Sig. 0.000)

Total Variance explained: 68.05%

**Table B.2:** Expectations towards four dimensions of marketing mix by using Exploratory

Factor Analysis in the Philippines of 18 items

| Factor name | Items | Factor 1 | Factor 2 | Factor 3 | Factor 4 |
| --- | --- | --- | --- | --- | --- |
| Product | Prod1 | 0.76 |  |  |  |
|  | Prod2 | 0.80 |  |  |  |
|  | Prod3 | 0.84 |  |  |  |
|  | Prod4 | 0.85 |  |  |  |
|  | Prod5 | 0.85 |  |  |  |
|  | Prod6 | 0.72 |  |  |  |
| Price | Price1 |  |  |  | 0.90 |
|  | Price2 |  |  |  | 0.88 |
|  | Price3 |  |  |  | 0.74 |
|  | Price4 |  |  |  | 0.76 |
| Place | Place1 |  | 0.85 |  |  |
|  | Place2 |  | 0.90 |  |  |
|  | Place3 |  | 0.89 |  |  |
|  | Place4 |  | 0.79 |  |  |
| Promotion | Prom1 |  |  | 0.81 |  |
|  | Prom2 |  |  | 0.86 |  |
|  | Prom3 |  |  | 0.90 |  |
|  | Prom4 |  |  | 0.86 |  |

Note: Extraction Method: Principal Component Analysis. Rotation Method: Varimax with Kaiser Normalization. Rotation converged in 5 iterations

KMO: 0.90; Bartlett's Test of Sphericity: Approximate Chi-square (5009.75; Sig. 0.000)

Total Variance explained: 77.28%

Total deleted items for both Bangladesh and the Philippines (2): Expectations toward food crop (Item 1_Visually appealing); Expectation toward Price (Item 3_Expensive)

**Decision criteria for deletion of items:** Corrected item-total correlation coefficients below 0.40 were considered for deletion,and whether the removal of the item could significantly enhance the total reliability of the questionnaire was considered by using Cronbach's alpha (Kuo et al., 2009)

**Table B.3:** The fit indices and analysis results for the measurement model of the marketing

mix expectations

| Fit indices | Recommended value | Result | |
| --- | --- | --- | --- |
|  |  | Bangladesh | The Philippines |
| χ^2^/df | <3.00 | 2.41 | 1.96 |
| GFI (goodness of fit index) | >0.90 | 0.92 | 0.93 |
| RMSEA (root mean square error of approximation) | <0.08 | 0.06 | 0.05 |
| RMR (root mean square residual) | <0.08 | 0.03 | 0.03 |
| NFI (normed fit index) | >0.90 | 0.92 | 0.95 |
| TLI (tucker-lewis index) | >0.90 | 0.94 | 0.97 |
| CFI (comparative fit index) | >0.90 | 0.95 | 0.97 |

**Table B.4:** Factor loadings and convergent validity results of the marketing mix expectations

| Constructs | Items | Factor loadings | | AVE | | Composite  Reliability (CR) | | Cronbach's alpha | |
| --- | --- | --- | --- | --- | --- | --- | --- | --- | --- |
|  |  | **BD** | **PHIL** | **BD** | **PHIL** | **BD** | **PHIL** | **BD** | **PHIL** |
| Product | Prod1 | 0.62 | 0.75 | 0.55 | 0.66 | 0.88 | 0.92 | 0.88 | 0.92 |
|  | Prod2 | 0.72 | 0.76 |  |  |  |  |  |  |
|  | Prod3 | 0.83 | 0.88 |  |  |  |  |  |  |
|  | Prod4 | 0.81 | 0.83 |  |  |  |  |  |  |
|  | Prod5 | 0.84 | 0.89 |  |  |  |  |  |  |
|  | Prod6 | 0.63 | 0.76 |  |  |  |  |  |  |
| Price | Price1 | 0.85 | 0.65 | 0.56 | 0.66 | 0.83 | 0.88 | 0.83 | 0.88 |
|  | Price2 | 0.67 | 0.65 |  |  |  |  |  |  |
|  | Price3 | 0.83 | 0.94 |  |  |  |  |  |  |
|  | Price4 | 0.61 | 0.95 |  |  |  |  |  |  |
| Place | Place1 | 0.74 | 0.88 | 0.72 | 0.79 | 0.91 | 0.94 | 0.91 | 0.94 |
|  | Place2 | 0.89 | 0.95 |  |  |  |  |  |  |
|  | Place3 | 0.89 | 0.93 |  |  |  |  |  |  |
|  | Place4 | 0.86 | 0.78 |  |  |  |  |  |  |
| Promotion | Prom1 | 0.58 | 0.74 | 0.52 | 0.71 | 0.81 | 0.91 | 0.80 | 0.91 |
|  | Prom2 | 0.60 | 0.83 |  |  |  |  |  |  |
|  | Prom3 | 0.82 | 0.94 |  |  |  |  |  |  |
|  | Prom4 | 0.83 | 0.85 |  |  |  |  |  |  |

Note: BD= Bangladesh; PHIL= The Philippines

**Table B.5:** Factors correlation and discriminant validity of marketing mix

|  | Bangladesh | | | | The Philippines | | | |
| --- | --- | --- | --- | --- | --- | --- | --- | --- |
|  | Product | Price | Place | Promotion | Product | Price | Place | Promotion |
| Product | **[0.74]** |  |  |  | **[0.81]** |  |  |  |
| Price | 0.34 | **[0.75]** |  |  | 0.45 | **[0.81]** |  |  |
| Place | 0.36 | 0.28 | **[0.85]** |  | 0.48 | 0.47 | **[0.89]** |  |
| Promotion | 0.50 | 0.29 | 0.40 | **[0.72]** | 0.41 | 0.31 | 0.27 | **[0.84]** |

Note: Diagonal elements (bold) show the square root of the average variance extracted (AVE). Off-diagonal elements show the shared variance.

**Table B.6:** Collinearity Test (Variance Inflation Factor)

|  | Bangladesh | The Philippines |
| --- | --- | --- |
| Product | 1.30 | 1.45 |
| Price | 1.14 | 1.33 |
| Place | 1.23 | 1.49 |
| Promotion | 1.33 | 1.19 |

**Reference**

Kuo, Y.-F., Wu, C.-M., & Deng, W.-J. (2009). The relationships among service quality, perceived value, customer satisfaction, and post-purchase intention in mobile value-added services. *Computers in Human Behavior*, *25*(4), 887-896.
